# Bridging science and spirituality: the intersection of religion and public health in the COVID-19 pandemic

**DOI:** 10.3389/fpsyt.2023.1183234

**Published:** 2023-05-19

**Authors:** Shahana Ayub, Gibson O. Anugwom, Tajudeen Basiru, Vishi Sachdeva, Nazar Muhammad, Anil Bachu, Maxwell Trudeau, Gazal Gulati, Amanda Sullivan, Saeed Ahmed, Lakshit Jain

**Affiliations:** ^1^Department of Psychiatry, Cornerstone Family Healthcare, Newburgh, NY, United States; ^2^Menninger Department of Psychiatry and Behavioral Science, Baylor College of Medicine, Houston, TX, United States; ^3^Developmental Behavioral Pediatrics, Dell Children's Medical Center, Austin, TX, United States; ^4^Adesh Institute of Medical Sciences and Research, Bathinda, Punjab, India; ^5^Baptist Health – UAMS Psychiatry Residency Education Program, North Little Rock, AR, United States; ^6^Department of Psychiatry, University of Connecticut, Farmington, CT, United States; ^7^Quinnipaic University, Hamden, CT, United States; ^8^Department of Psychiatry, Rutland Regional Medical Center, Rutland, VT, United States; ^9^Connecticut Valley Hospital, Middletown, CT, United States

**Keywords:** COVID-19, religion, spirituality, public health, pandemic, misinformation, vaccine hesitancy and refusal, conspiracy

## Abstract

**Background:**

The COVID-19 pandemic has had global impacts on social interactions and religious activities, leading to a complex relationship between religion and public health policies. This article reviews impact of the COVID-19 pandemic on religious activities and beliefs in relation to the spread of the virus, as well as the potential of religious leaders and faith communities in mitigating the impact of the pandemic through public health measures and community engagement.

**Methods:**

A literature review was conducted using PubMed and Google Scholar, with search terms including “religion,” “COVID-19,” “pandemic,” “coronavirus,” and “spirituality.” We included English articles published between January 2020 and September 2022, focusing on intersection of religion and COVID-19.

**Results:**

We identified two main themes emerging, with the selected 32 studies divided in 15 studies focused on the relationship between religious practices, beliefs, and the spread of COVID-19, while 17 studies explored the role of religious leaders and faith communities in coping with and mitigating the impact of COVID-19. Religious activities were found to correlate with virus spread, particularly in early days of the pandemic. The relationship between religiosity and adherence to government guidelines was mixed, with some studies suggesting increased religiosity contributed to misconceptions about the virus and resistance to restrictions. Religious beliefs were also associated with vaccine hesitancy, particularly conservative religious beliefs. On the other hand, religious leaders and communities played a crucial role in adapting to COVID-19 measures, maintaining a sense of belonging, fostering emotional resilience, and upholding compliance with public health measures. The importance of collaboration between religious leaders, institutions, and public health officials in addressing the pandemic was emphasized.

**Conclusions:**

This review highlights the essential role of religious leaders, faith-based organizations, and faith communities in promoting education, preparedness, and response efforts during the COVID-19 pandemic. Engaging with religious leaders and communities can improve pandemic control and prevention efforts. Collaboration between religious leaders, governments, and healthcare professionals is necessary to combat vaccine hesitancy and ensure successful COVID-19 vaccination campaigns. The insights from this review can guide future research, policy development, and public health interventions to minimize the impact of the pandemic and improve outcomes for individuals and communities affected.

## 1. Introduction

The COVID-19 pandemic has impacted nearly every aspect of our lives, its implications even reaching our religious activities, which remains an important topic of debate and ongoing research ranging from social distancing at religion functions to vaccination acceptance among congregations. A significant toll has been taken on traditional human connections such as these, and this has forced all stakeholders to adopt innovative approaches to address religious gatherings' emergent issues. Whether holding virtual meetings or deploying contact tracing apps, individuals and organizations alike have adopted creative ways to continue communing with one another while trying to keep the risk of transmission low. Historically, religion has served a crucial role in shaping public health outcomes during times of crisis—consider the Ebola epidemic, pandemic influenza, and ongoing worldwide health concerns such as HIV/AIDS (1). Indeed, religion's influence has appeared before us in beneficial, and at times, detrimental ways during these emergencies (1, 2). Positive and negative impacts have stemmed from religious gatherings and rituals due to the extent which religious leaders have adhered to established guidelines. In the case of COVID-19, while some religious groups have been praised for their adherence to public health precautions, others have received criticism for disregarding limitations and thus contributing to the virus's spread. This latest pandemic highlighted the role of religious institutions and practices in either curbing or accelerating viral spread, as well as their contribution to public health efforts to control previous pandemics, such as the Spanish flu and H1N1.

The COVID-19 pandemic led to changes in religious practices and rituals, with some communities resisting or outright defying restrictions suggested by their national or local governments or scientific communities. In turn, this had the negative effect of contributing to viral spread. Unfortunately, these negative cases pitted religion against evidence-based science, with the former seeing its adherents clinging to their religious faith for protection instead of listening to scientific advice (1). Meanwhile, in the positive sense, other religious communities successfully followed both religious guidance and scientific recommendations to reduce the risk of viral spread.

To better understand the interplay of religion and public health amid the COVID-19 pandemic, several global studies have explored the ways religion has influenced individuals and communities during this crisis. One study revealed how the pandemic forced religious leaders to redesign mosque worship and how Muslims adapted their practices (3). Another study in Israel revealed how ultra-Orthodox Jewish communities experienced a significantly higher rate of COVID-19 infections due to factors such as overcrowding, distrust of state authorities, and resistance to social-distancing orders. The rapid spread of the virus in such religious communities increased tensions and raised questions about the balance between religious practice and public health (4).

Studies from global regions as diverse as Ghana, Poland, and Malaysia have explored the impact of COVID-19 on religious communities, psychospiritual gatherings, and other religious practices; as well as the role of religious expression in coping with pandemic-derived stress. In Ghana, Osei-Tutu et al. (5) explored religious leaders' views on the impact of COVID-19 as it related to restrictions placed on their congregants' wellbeing. The study found that people suffered a plethora of psychospiritual effects due to the pandemic, such as a decline in spiritual life, a sense of loss of fellowship and community, financial difficulties, anxiety over childcare, and fear of infection (5). Osei-Tutu's study revealed how religious leaders positively intervened by delivering sermons on hope, faith, and repentance, with some going so far as to sensitize their membership to topics such as health hygiene and COVID-19-related stigma. In Poland, Sulkowski et al. (6) investigated the impact of the pandemic on that country's religious life, finding that some churches either limited or entirely suspended their traditional community-based religious life in light of the pandemic, seeking to reduce risk of viral spread while maintaining contact with and among believers via modern technology (6). A Malaysian study by Ting et al. (7) investigated several pandemic-related variables, such as illness perception, stress levels, and religious expressions of major religious groups (7). Ting et al. (7) study notably reported that religious expression carried a negative relationship with stress levels, highlighting the importance of religion's role in shaping responses to public-health emergencies, particularly in communities where religion serve a significant role in people's lives. Taken together, these studies confirm the important, even primary role religion can play in shaping responses to public health emergencies (5–7).

Researchers from other countries such as Colombia, South Africa, and the United States, have examined the roles of hope, religious coping, and community organizations in promoting wellbeing during the COVID-19 pandemic. Counted et al. (8) examined these roles and their wellbeing effects in Colombia and South Africa, revealing that hope was positively associated with wellbeing and that the relationship between hope and wellbeing was itself moderated by religious coping. When hope was low, the researchers found, wellbeing trended higher when positive religious coping was high and negative religious coping was low (8). This study highlights the importance of considering the role of religious leaders and their support in addressing the psychospiritual impacts of the pandemic, particularly in communities where religion plays a significant role in people's lives, as other studies have concluded. In the United States, Weinberger-Litman's (9) study examined anxiety and distress among members of the first community in the USA to be quarantined due to the COVID-19 pandemic, a community of Orthodox Jews (9). The study found that community organizations were trusted more than any other source of COVID-19-related information and played a vital role in promoting the wellbeing of their constituents by organizing support mechanisms such as the provision of tangible needs, social support, virtual religious services, and dissemination of virus-related health information. In their conclusions, these studies supported the findings of the mentioned prior ones (8, 9).

Similar studies conducted in Portugal, Bosnia and Herzegovina, and New Zealand have explored the roles of spiritual-religious coping, religious freedom restrictions, and worship adaptations during the COVID-19 pandemic (10–12). Prazeres et al. (11) examined the impact of spiritual-religious coping on fear and anxiety related to COVID-19 in Portugal's healthcare workers, finding that religiosity was not a significant factor in reducing coronavirus-related anxiety, and that higher levels of hope and optimism along the spirituality scale were associated with less anxiety (11). Begović (10) found that religious communities in Bosnia and Herzegovina displayed varying responses to pandemic restrictions on religious freedom imposed by state regulations (10). Here, some communities willingly agreed to the restrictions placed on their religious guidelines and practices, while others struggled to agree. Despite their differences, the researcher found, all communities were able to find support in their religious laws and theological views, which emphasized the value of human life and the importance of caring for their community's wellbeing (10). In New Zealand, Oxholm (12) reported that the COVID-19 pandemic caused religious communities to review their worship practices and prioritize community welfare and pastoral care for the elderly and vulnerable (12). To that end, congregations shifted to virtual worship. In this case, the challenges of mitigating transmission risk, social distancing, and providing welfare overlapped (12).

COVID-19 indeed caused significant global upheaval, leading to quarantines and a rising death toll. With healthcare professionals plying science to control the virus, religious organizations and psychospiritual groups provided solace while, with a few exceptions, also contributing to the recommended protective measures, such as social distancing and the cancellation or conversion of large gatherings in some faiths (13). The exceptions included the Islamic State, which regarded the pandemic as divine retribution; and Feng shui practitioners, who attributed it to an imbalance of elements in the Year of the Rat (14). It remains notable that major religious gatherings were identified as significant clusters of viral spread in Singapore, Malaysia, and South Korea (14).

For complying religious groups, the pandemic prompted a transition from in-person religious communities to virtual congregations, challenging conventional notions of belonging and participation (15), This transformation required embracing digital platforms for live-streaming of services, Zoom baptisms, and Skype confessions, etc. (15, 16) while less-compliant religious groups resisted change. Additionally, religious leaders here offered explanations and comfort to their congregations during uncertain times, highlighting the resurgence of religion and spirituality in the face of a global crisis (14).

A survey study by Seryczynska et al. (17) explored the role of religious capital in coping during the COVID-19 pandemic in four European countries: Spain, Italy, Poland, and Finland. Their results revealed that religious capital indeed can impact individuals' coping strategies, but its dynamics, the ways it does so, are complex (17). This survey's results provide a better understanding of the role of religious capital in helping people cope with harsh circumstances.

While the scientific community has largely come together in controlling the spread of the coronavirus, primarily through advising mask wearing, social distancing, and developing vaccines, its pandemic-curtailing efforts have been hampered by various factors, oftentimes religious gatherings. Such gatherings provide an essential role in society, but governments, the scientific community, and healthcare entities worldwide have experienced pushback from certain religious entities regarding their advised pandemic-response measures.

The present paper reviews the impact of the COVID-19 pandemic on lives around the world, including the responses by the respective public health authorities, the associated factors that dictated the outcome of those responses, and the implications of the intertwined nature of religion, public-health policy, and social responsibility. The researchers subsequently investigate two main intersections where religion met public health during the COVID-19 pandemic: first, the connections between religious practices, beliefs, and the spread of the virus, including both the positive and negative consequences of religious activities; and second, the role of religious leaders and faith communities in coping with and mitigating the impact of COVID-19. In doing so, we hope to offer a unique multidisciplinary perspective on the complex interplay between religious practices and public-health outcomes, emphasizing the importance of taking a balanced, holistic approach to mitigating—or preventing—public health crises. By synthesizing this intersectional area's existing research, we can not only contribute to the related literature by providing a better understanding of the intersection of religion and public health crises, but also identify any potential gaps warranting future research.

## 2. Method

We conducted a structured and systematic literature search on PubMed and Google Scholar using keywords such as “religion,” “COVID-19,” “pandemic,” “coronavirus,” and “spirituality” to study the intersection of religion and the COVID-19 pandemic. Our search was conducted from January 2020 to March 2023. We included peer-reviewed articles, published in English language, primarily observational studies, cross-sectional studies, surveys, and systematic reviews. We excluded case reports, case series, non-English papers, papers not directly related to the topic, papers with data that was difficult to extract, and unpublished papers. The articles must focus on one or both of the following aims: investigating connections between religious practices, beliefs, and the spread of the COVID-19 virus, including both the positive and negative consequences of religious activities; and exploring the role of religious leaders and faith communities in coping with and mitigating the impact of the COVID-19 pandemic.

**Figure 1:**
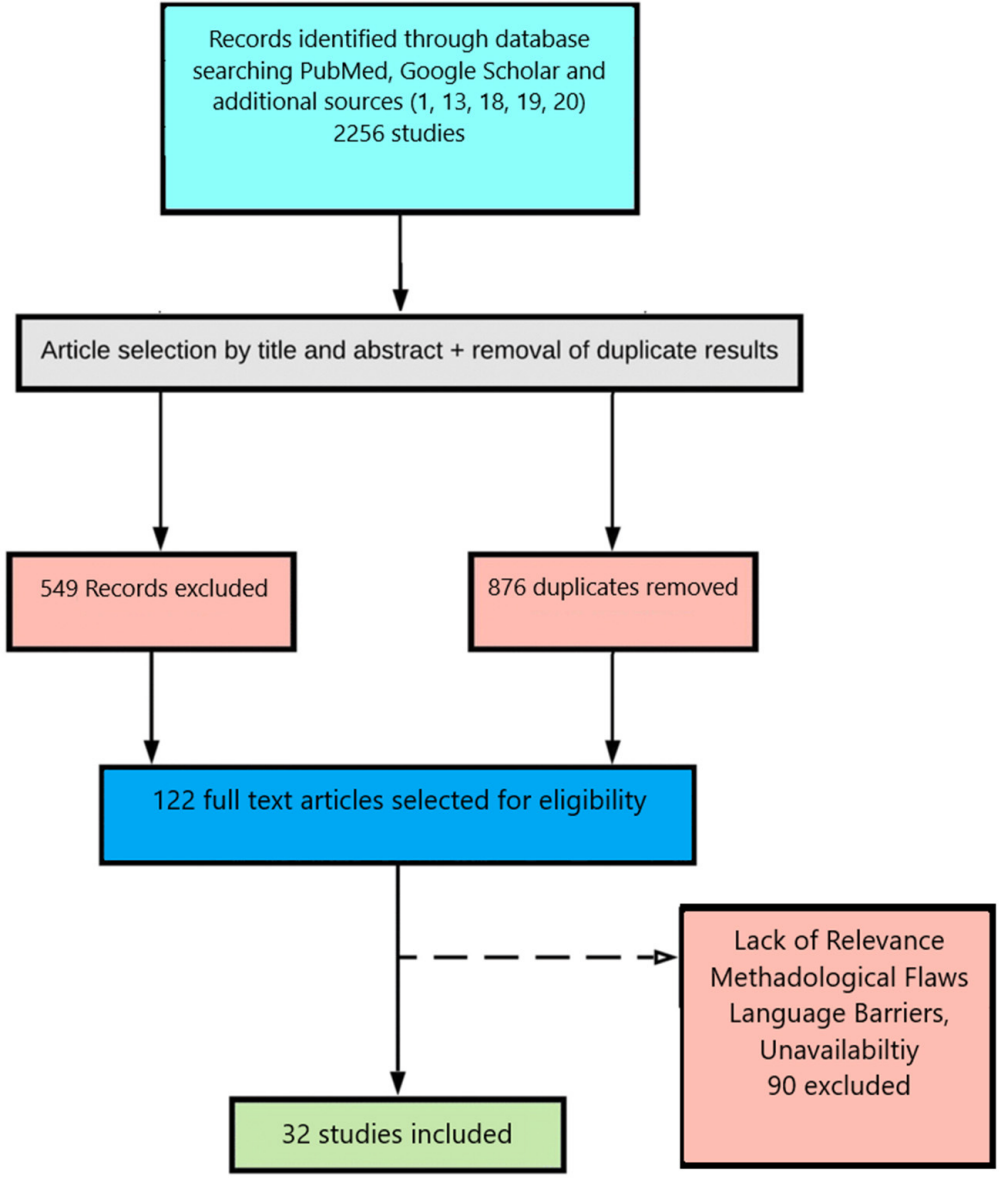
Search strategy.

Initially, we identified 2,256 papers through our search strategy, which we then narrowed down to 876 by removing duplicates. After screening the titles of these papers, we included 327 citations for abstract screening. During abstract screening, we excluded 549 citations based on our inclusion criteria, which focused on studies that provided insights into the role of religion and religious activities in the context of the COVID-19 pandemic. The remaining 122 papers were reviewed for eligibility by SA and LJ, and any disagreements were mediated by a third reviewer (SA). Additionally, we employed a snowballing technique whereby we identified and selected review articles on our topic of interest (1, 13, 18–20), and then used their reference lists to further identify relevant studies. This approach helped us to expand our search results and ensure that we did not miss any important studies that were relevant to our topic. We have included a study selection flow diagram (Figure 1) to illustrate the process of identifying, screening, and selecting articles for our review. This diagram provides a transparent representation of our literature search and selection process.

## 3. Results

### 3.1. Description of studies included

32 full-text articles were identified that met our inclusion criteria for this literature review. We categorized these articles into two tables based on their focus: Table 1, which captures studies investigating the relationship between religious practices, beliefs, and the spread of COVID-19, and Table 2, which explores the role of religious leaders and faith communities in coping with and mitigating the impact of the pandemic. We acknowledge that the studies included in our review come from various research designs, and our aim was to provide a comprehensive overview of the existing literature on the topic rather than a strict synthesis of the findings.

**Table 1:** Studies focusing on the role of religious practices and beliefs in the spread of COVID-19.

**Study**	**Author(s)/Source**	**Religion**	**Year**	**Objectives, observations, study highlights, and key findings**
1.	Quadri and Padala (21)	Hinduism	2021	• The study focuses on individuals practicing Hinduism and their deeply rooted historical traditions, including pilgrimages and religious gatherings. • Study's focus is on the significance of *Kumbh Mela*, a Hindu religious festival that takes place in India, and it's religio-socio-cultural aspects in the context of COVID-19 and it's impact. • The study outlines the various ceremonies performed by the devotees during the *Kumbh Mela*, and the potential of these ceremonies to contribute to the COVID-19 outbreak in India. • Authors proposed several strategic interventions that can be followed by religious leaders, government officials, elected leaders and to avert the *Kumbh Mela* from contributing to a public health emergency. these steps include - ° Restricting the number of participants and preventing the sick and the vulnerable elderly from participating. ° Registering participants and using technology (Drones, GPS tracking etc.) to monitor and trace devotees. ° Spreading information about COVID-19 by working together with religious leaders to promote COVID-19 guidelines (mask wearing, hand sanitization, etc.). ° Providing alternative pilgrimage sites. ° Develop a comprehensive response strategy. ° Improving healthcare infrastructure and expanding quarantine facilities.
2.	Quadri (22)	Islam, Christianity, Judaism, Sikhism, Hinduism	2020	• The study examines the implications of religious gatherings during the pandemic. • Authors present various examples from different religions and countries, illustrating how religious congregations contributed to the spread of COVID-19 and how suspending these gatherings led to a slower spread of the virus. • The study highlights how countries that suspended religious gatherings early had lower incidences of COVID-19 infections. • The study highlights the importance of collaboration between clergy and government in suspending religious gatherings and creating contingency plans for infectious epidemics.
3.	Al-Rousan and Al-Najjar (23)	Islam (Shi'ite sect) and Judaism	2020	• The study examines various Islamic sects, focusing on their traditional pilgrimages and religious gatherings during the COVID-19 pandemic. • Authors utilized hierarchical clustering principles to trace COVID-19 infections in the Middle East and link the spread of COVID-19 to human mobility for religious reasons (visiting holy places, pilgrimage etc.). • Key findings: Jewish pilgrims may have spread COVID-19 to Israel via religious rituals as well. • Human mobility, tourism, and visiting religious sites are the main causes of COVID-19 spread in various countries. • The study identified human mobility, tourism, and visiting religious sites are the main causes of COVID-19 spread in various countries. • Authors presented some solutions such as the closure of borders between Gulf countries, Lebanon, and Iran to prevent further human mobility and exposure to people who traveled to Gulf countries.
4.	Kim et al. (24)	Shincheonji Church of Jesus	2020	• Authors utilized data provided from Korea's Center for Disease Control and Prevention, Department of Public health and news reports to establish time delay from illness onset to COVID-19 confirmation in COVID-19 cases linked to the Shincheonji religious group. • This paper discusses the beliefs and religious practices of the Shincheonji religious group, and how they conflict with COVID-19 guidelines. • The study highlights how Shincheonji religious group's practices contributed to many of their members being infected with COVID-19. • The study discusses the legal steps enacted by the Korean Government to tackle this challenge.
5.	Linke and Jankowski (25)	Multiple	2022	• The study analyzes data from 47 countries in World Values Survey Wave 7. • The study aims to examine the relationship between the COVID-19 pandemic situation in the country and internal/external indices of religiosity and religious fundamentalism. • Results show that countries with more residents attending religious services had more COVID-19 cases and more deaths. • Authors noted an observation that fewer COVID-19 tests were conducted in countries with higher percentages of the population declaring belief in God and trust in religion over science.
6.	Taragin-Zeller et al. (26)	Judaism (Haredi Judaism)	2020	• The study focuses on COVID-19 related decision-making by the Ultra-Orthodox (Haredi) Jews in Israel. • Authors found that Haredi men and women made COVID-19-related decisions based on religious and medicine-related rationalizations. • The study highlights the need to develop better science communication models and creative strategies to tailor their message to minorities.
7.	Hill et al. (27)	Christianity	2020	• Authors investigated how religiosity affects social isolation and adherence with stay-at-home guidelines by evaluating the average distance traveled per day using location of people compiled by using mobile phone, Wi-Fi, and GPS data. • Key findings: People in states with higher religiosity traveled longer distances and thus had higher average mobility scores in the period Feb 24 to March 2 • The study found that in highly religious states, stay-at-home directives have a lesser impact on people's mobility. • Reductions in travel during the pandemic were smaller in more religious states. • Religious states were more resistant to stay-at-home orders. • The findings emphasize that non-compliance with COVID-19 restrictions in religious communities increases the risk of contracting and spreading the disease over larger distances.
8.	Boguszewski et al. (28)	Christianity	2020	• Authors utilized a structured questionnaire to explore how COVID-19 affected religiosity in Poland. • Results shows that 21.3% reported devoting more time to prayer than before COVID-19-related restrictions were enacted. • Key findings: People who were religiously involved prior to the pandemic also reported an increase in religious activity and reported increased satisfaction with their lives. • People who reported themselves as minimally involved in religious activities experienced more anxiety about losing their job. • There was a positive correlation between increased religiosity and misconceptions about the coronavirus. • Individuals with higher religious activity showed less compliance with government restrictions, had more COVID-19 misconceptions, relied on informal information sources, and expressed more conspiracy theories, despite wearing masks. • One unique observation was made in this study, that people who pray more may be less fearful of the epidemic than others.
9.	Levin J and Bradshaw (29)	Christianity	2022	• Data from the 2021 Gallup Values and Beliefs of the American Public Survey was used. • Key findings: The study reported that skepticism and vaccine hesitancy were strongly associated with one's political preference and conservative religious beliefs. • Other findings were having a loved one suffer from COVID-19 did not significantly change the respondents' beliefs. • The researchers pointed out the potential impact of these findings on vaccine acceptance and public health efforts.
10.	Perry et al. (30)	Christianity	2020	• The research delves into the concept of Christian nationalism as a separate entity from religion, with the authors proposing a hypothesis and analyzing data from three waves of the Public and Discourse Ethics Survey. • Key findings: The study reported that Christian Nationalism is positively linked to more frequent incautious behavior regarding COVID-19 and negatively associated with taking necessary precautions. • Religious commitment was the leading factor that could predict if a person will avoid taking precautionary measures in order to prevent COVID-19 spread. • Religious commitment promotes prosocial values and behaviors once Christian nationalism is factored out.
11.	Perry et al. (31)	Christianity	2021	• Authors analyzed date from the Public and Discourse Ethics Survey to explore the relationship between xenophobic and racist perspectives on COVID-19 and White nationalism. • Key findings: An association between the aforementioned perspectives and White nationalism was found to persist even after accounting for various sociodemographic, religious, and political factors. • Christian nationalism was found to be a strong predictor of White Americans holding racist views compared to other races. • More than 55% of the white sample tested above the mean on the white nationalism scale, indicating that this is not a fringe movement.
12.	Lee et al. (1)	Islam, Judaism, Christianity (Catholicism, Shincheonji Church of Jesus, etc.)	2022	• Review of 58 articles examining the role of religious communities in COVID-19 spread. • Key findings: Religious gatherings and practices played a key role in COVID-19 spread in the initial days of the pandemic. • Vaccine refusal observed due to religious reasons. • Religious institutions collaborated with government authorities to spread scientific knowledge about COVID-19 and helped to address vaccine hesitancy. • Dual role of religion: both accelerating and mitigating the spread of COVID-19.
13.	Widiyanto et al. (3)	Islam	2020	• The study explores challenges of handling COVID-19 in Indonesia. The challenges include misinformation propagated by social media. • Observations include conservatives embracing COVID-19 conspiracy theories and religious conservatism leading to rejection of government guidelines. • Author recommends cultivating a “new spirituality” that aligns with science and knowledge to combat the pandemic. • Authors proposes developing a “Muslim Knowledge Culture” to promote adherence to public health guidelines and overcome misinformation.
14.	Lorea (14)	Christianity, Hinduism, Islam, Judaism	2020	• The study examines how religious leaders had diverse responses to COVID-19 pandemic as they tried to make sense of the COVID-19 pandemic using religious knowledge. • In the article, the author explores how some religions have adapted to using digital devices to organize ceremonies and rituals, while others have resisted this change. • Key findings: Some communities modified their rituals to comply with COVID-19 guidelines (e.g., using disposable q tips for holy water, sharing *prasad* on platter), others refused to change their ways (insisting to kiss the Torah, touching Pir's shrine, drinking from shared spoon etc.) • Some religious leaders promoted religion-based explanations of COVID-19 (accumulation of negative *karma*) and even predict that pandemic will subside by a certain day. • The study emphasizes the influence of religious leaders in interpreting and rationalizing religious beliefs, while underscoring the resurgence of religion and spirituality for comfort and predictions, urging social scientists to address these shifts.
15.	Pirutinsky et al. (32)	Judaism	2020	• The study focused on the American Orthodox Jewish community in the states of New York and New Jersey. • Key findings: High levels of religiosity increased the risk of direct exposure to COVID-19. • Highly religious participants felt low levels of distress caused by the COVID-19 pandemic. • Highly religious participants showed high levels of compliance with government restrictions. • Increased trust in God and intrinsic religiosity were observed as forms of positive religious coping. • Utilizing positive religious coping led to better mental health.

**Table 2:** Exploring the role of religious leaders and faith communities in coping with and mitigating the impact of COVID-19.

**Study**	**Author(s)/Source**	**Religion**	**Year**	**Objectives, observations, study highlights, and key findings**
1.	Frei-Landau (33)	Judaism	2020	• Commentary discussing innovative adaptations to traditional rituals by Israeli Jewish community for social distancing compliance. • The adaptations were made in order to prevent the spread of COVID-19. • The study suggests that these alternative mechanisms may help maintain a sense of belonging and foster emotional resilience.
2.	Impouma et al. (34)	Unknown	2021	• The study is a retrospective observational cross-sectional analysis that examines whether COVID-19 preparedness and response strategies were influenced by experience gained during previous Ebola virus disease (EVD) outbreaks. • Primary focus on countries including Guinea, Liberia, and Sierra Leone. • Authors found that the rapid implementation of readiness and response measures (within weeks in COVID-19 pandemic) could be attributed in part to the experience gained from the EVD outbreak of 2014-2016. • Response measures included suspensions of flights and schools, restrictions on internal movement and mass gatherings, mask wearing and mandatory testing. • The high Case Fatality Rate (CFR), low number of tests per 10,000 population and delays in reporting and confirming cases indicated that the healthcare system was still underfunded and fragile.
3.	Williams et al. (35)	Buddhism, Judaism, Islam, Hinduism, Christianity (Catholicism, Protestant, and Unity sect)	2020	• Qualitative analysis based on interview transcripts with clergy in Colorado and North Carolina. • The interviews were conducted from October 2018 to September 2019. • Authors used a grounded theory approach to identify themes in the transcripts. • Key findings: Authors found that most clergy members held positive views on vaccines. • The clergy were open to the idea of vaccine advocacy and desired help from experts to address vaccines in local settings.
4.	Orlandi, Febo et al. (36)	Multiple	2022	• The study was conducted in 22 European countries. • The study identified an overall negative and significant association between country-level religiosity and vaccination rates. • Key findings: In countries where Roman Catholics are the majority religious group, the association was reversed. • Roman Catholics displayed a positive association, likely due to the vocal acceptance of vaccines by the Pope.
5.	Yezli and Khan (37)	Islam	2021	• Authors explored the importance of the cessation of Umrah in February 2020 by the Kingdom of Saudi Arabia (KSA). • The study highlights the importance of temporarily closing places of worship and suspending religious gatherings to stop the spread of COVID-19. • Authors examined the role of religious leaders and institutions in promoting compliance with public health measures to prevent the spread of COVID-19. • The study emphasizes the need for religious leaders and institutions to work collaboratively with public health officials to address the COVID-19 pandemic.
6.	Yezli and Khan (38)	Islam	2020	• Authors explored the challenges faced by the Kingdom of Saudi Arabia (KSA) administration. • Key findings: The KSA administration enacted strict COVID-19 social distancing guidelines despite political, economic, social, and religious challenges. • Authors examined the challenges and complexities of implementing such measures in a religiously conservative society. • The study highlights the importance of taking bold steps to halt the spread of COVID-19 in the future.
7.	Gautret and Al-Tawfiq (39)	Islam	2020	• The letter to the editor compares the 2020 Olympics and Hajj Pilgrimage. • Authors discuss whether these events should be suspended. Authors use multiple reasons to support their argument. • The suspension of Umrah is highlighted as a positive step in controlling the spread of COVID-19.
8.	Waitzberg et al. (40)	Judaism, Islam	2020	• Authors explored steps taken by the Government of Israel in tackling COVID-19. • Israel's government tailored COVID-19 protocol for religious minorities, such as Ultra-Orthodox Jews (12% of population). • Key findings: The Ultra-Orthodox Jews had lower access to healthcare due to several barriers and did not utilize regular media or methods of communication. They also live in tight knit communities that follow instructions of their own leaders. • The government-built trust among Orthodox Jewish leadership, recruited aid and charity networks, and communicated the importance of restriction measures. • Despite communications, Ultra-Orthodox schools and synagogues stayed open 1-2 weeks after government mandated shutdowns. Once the leaders communicated the instructions to the community, the community complied quickly. • As the Pandemic worsened, Army was called in to help establish quarantine, help provide food and resources to people and these strict conditions were continued in the Passover holiday. • Authors concluded that managing COVID-19 spread in less accessible groups requires stricter social distancing and cooperation with religious leaders; similar steps should be taken for Arab minorities facing language barriers, poverty, and upcoming Ramadan holiday.
9.	Thompkins et al. (41)	Christianity	2020	• Authors documented responses to 15-min videos produced as part of Project Trust (PT). • The videos included pastors, public health officials, and mental health providers discussing their individual COVID-19 experiences. • Identified four core themes for addressing the needs of at-risk African Americans during the pandemic: ritual disruption, guideline adherence, trauma, and culture and trust. • Themes represented challenges faced by African American churches. • Discussed four action avenues for addressing these challenges and moving forward.
10.	Galang (42)	Christianity, Hinduism	2021	• The correspondence was written in response to A letter by Corpuz GC (2021)(40). • Author discussed the moral obligation of religious leaders. • Religious leaders have a responsibility to accept the findings of science. • Religious leaders should not disseminate COVID vaccine-related misinformation to their followers. • Dissemination of misinformation undermined people's faith in science and the vaccine.
11.	Osei-Tutu et al. (5)	Christianity	2021	• Authors interviewed 15 religious leaders from Ghana following a ban on religious gatherings enacted by the government. • Key findings: The leaders identified several impacts on their congregants, including financial challenges, disruption to childcare/training, fear of infection, decline in spiritual life, and loss of fellowship and community. • The leaders approached the challenges using several methods, including instilling hope, and sustaining faith, sermons on repentance, and implementing hygiene protocols and COVID-19-related stigma sensitization.
12.	Sulkowski et al. (6)	Christianity	2020	• Authors conducted in-depth interviews with 12 priests and pastors of churches in Poland that were conducted from the day the Government announced the COVID-19 epidemic in Poland. • Key findings: Many were ready to modify their worship practice, while others suspended or reduced them depending on ecclesiology. Some even asked for more drastic measures. • Some churches still considered the possibility of maintaining services while following precautions, others were more decisive in suspending them. • The clergy were not willing to change worship itself (like stopping communion distribution) but were open to modifying it by giving communion wafer in hand instead of mouth and pouring wine in individual goblets. • Some clergy saw the COVID-19 pandemic because of sin/human degradation, they all called for helping the fellow human, the vulnerable elderly and healthcare workers. • Many clergy utilized the internet to share religious information, sermons, and meditations. While some used the state media, others had their own websites and some utilized Facebook and YouTube. Some did not use social media. • The Churches cooperated to hold joint services and invited professionals to disburse information on the COID-19 pandemic. • Some clergy endorsed the state's decisions while others saw themselves as a regular person following the law for their community.
13.	Weinberger-Litman et al. (9)	Judaism (Modern Orthodox Jewish)	2020	• Online questionnaire-based study conducted in March 2020 among Modern Orthodox Jewish community in NY and CT, the first quarantined religious community in these states. • Key findings: Significantly high level of religious commitment observed (56.8% reporting religion as “very important” and 25.7% as “center of my world”). Religious community leaders organized various types of support for members as follows: • Tangible supports food delivery. • Social support like calling the elderly to check up on them. • Information support about the quarantine • Religious support via online religious meetings and rituals
14.	Begović (10)	Christianity (Orthodox and Catholic), Judaism, and Islam	2020	• Examined COVID-19 restrictions on religious activity in Bosnia and Herzegovina, with varied responses from religious communities: The responses of the religious community were as follows: • Key findings: The Islamic community utilized their central leadership to communicate to their members to strictly follow state guidelines and adapted its religious activity to COVID-19 restrictions. • Religious rituals like Friday prayer and Ramadan were conducted with close adherence to COVID-19 restrictions. • The Catholic Church exhibited a decentralized response, allowing dioceses to interpret guidelines individually, resulting in varied compliance among leaders – some followed guidelines and granted forgiveness for non-attendance at masses, while others publicly opposed restrictions and demanded in-person attendance. • The Orthodox church showed less determination in limiting church activity and following guidelines, some dioceses agreed to stick to state regulations while other chose to ignore them, some blamed the people for attending religious ceremonies in violation of state restrictions. • The Jewish Community suspended religious gatherings, and a network of volunteers was created to help the elderly with food and medicine. • Authors concluded that while all religions could justify restrictions through religious law and theology, some struggled to reconcile state regulations with religious autonomy.
15.	Oxholm et al. (12)	Christianity, Buddhism	2020	• Authors conducted interviews and analyzed Facebook and online news media, examining the response of religious leaders to COVID-19 restrictions both before and after level 4 lockdown. • Key findings: Many leaders endorsed internet resources to virtually conduct religious worship. • Some communities made this transition seamlessly, but others found certain aspects like shared communion difficult. • Many leaders recognized that religious practices increased the risk of COVID-19 transmission, leading to event cancellations, but individual concerns persisted, such as a shared communion cup causing a transmission, skepticism about the threat, and lockdown violations by door-to-door proselytizing. • Religious community-run food banks also adapted their practices to comply with COVID-19 guidelines. • The study concluded that since the event of communion cup sharing, religious communities took steps to address COVID-19 transmission in agreement with the state response to prioritize the welfare of their communities.
16.	Ting et al. (7)	Christianity, Islam, Hinduism and Buddhism	2021	• Authors conducted a 10-15 min long online survey using a mixed method research design with cross sectional data as part of a larger national project. Qualitative analysis revealed Religion as a double-edged sword with the following findings. • Key findings: Higher levels of religiosity (both internal and external) led to lower level of stress during the lockdown. While internal religiosity led to decreased stress from the loss of control, external religiosity may lead to increased stress of being infected, likely during religious gatherings. • Lockdown and Governmental policies affected the religions differently, with Muslim groups reporting the highest confidence as the COVID-19 information was printed in Malay while Buddhism groups were affected the most due to lockdowns. • Authors offered recommendations: Religious communities can be encouraged to participate in prevention of COVID-19 pandemic. Religious leaders should be engaged in planning such programs.
17.	Counted et al. (8)	n/a	2020	• A study on Colombians and South Africans during homebound restrictions due to COVID-19 revealed that positive religious coping was associated with higher levels of wellbeing in both populations. • Authors reported as association between trait hope and wellbeing was moderated by positive religious coping among Colombians and negative religious coping among South Africans.

## 4. Narrative synthesis

### 4.1. The relationship between religious practices, beliefs, and the spread of COVID-19

The role of religious gatherings, ceremonies, and practices in contributing to the spread of COVID-19 has been noted in published literature. Researchers reported that these types of events played a significant role particularly in the pandemic's early days (1, 22–24). Though a strong correlation existed between religious activities and viral spread, it is worth noting that the study by Lee et al. (1) emphasized that religious activity could both accelerate and mitigate COVID-19. This illustrates religion's dual potential effects on public health crises. The relationship between religiosity that adhered with guidelines recommended or mandated by various government remains unclear. Some studies have found people in more religious areas or with higher levels of religiosity were less likely to comply with social distancing and stay-at-home orders (27, 32). Other studies have found some correlation between increased religiosity and misconceptions about the virus, as well as resistance to government mandated restrictions (28).

Regarding vaccination acceptance, religious beliefs had association with vaccine hesitancy, particularly when conservative religious beliefs linked with skepticism toward vaccines and overall public-health initiatives (1, 29).

The pandemic saw various religious leaders from various countries facing unique challenges in caring for their communities. Studies showed that effective communication and collaboration with public-health authorities or local governments remained vital in promoting adherence to guidelines and dismantling misinformation (3, 26, 43). The pandemic has also caused changes in religious practices. While some individuals have turned to religion for comfort and support during these uncertain times, others have experienced a crisis of faith or questioned their beliefs (14, 28).

Given the mixed findings on the relationship between religiosity and compliance with COVID-19 guidelines, it is important for researchers to continue examining it.

### 4.2. The role of religious leaders and faith communities in coping with and mitigating the impact of COVID-19

The published literature shows the impact of the pandemic on various religious communities and the challenges they faced, whether financial, childcare disruption, fear of infection, or loss of fellowship (5, 41).

Several studies revealed the importance of religious leaders and communities adapting to COVID-19 measures to maintain a sense of belonging and foster emotional resilience among their congregations (33, 35, 37). Some studies highlight how religious communities adjusted their traditional religious ritual and practices, ultimately complying with social distancing guidelines and safety measures to prevent viral spread (6, 12, 36). Other studies revealed the importance of collaboration between religious leaders, institutions, and public health officials in addressing the pandemic (37, 42). Still others revealed the important role religious leaders and institutions have played in upholding compliance with public-health measures and providing various support services (9, 10). Overall, most published studies underscore the need for religious leaders to accept scientific findings and resist disseminating COVID-19 and vaccine-related misinformation (42).

## 5. Discussion

The evaluated studies highlight the complex relationship between religion, religiosity, and the COVID-19 pandemic. Religious gatherings and activities played significant roles in the spread of COVID-19 across the world, as evidenced by the reviewed published studies (1, 3, 22–25, 27, 29–32). The literature shows the role of religious activity in amplifying the spread of COVID-19, through non-wearing of masks, non-adherence to social distancing, and at times through the promotion of misinformation. While some studies have suggested religion as a risk factor for contracting COVID-19; other studies identify religion as a positive source of coping and resilience (1, 26, 32).

The ravages of the COVID-19 pandemic led to existential crises in many, with some religious believers finding meaning by leaning into apocalyptic narratives, which some of the secular also did (20). Viewing the pandemic as an act of a superior being, certain religious leaders and organizations refused to change their group rituals and ceremonies. Several pastors expressed the belief that only God would decide when someone died and not the government, and thus refused to stop holding packed church services (44). Such religious defiance and harmful-belief promotion ultimately led to a rejection of government recommended or mandated COVID-19 guidelines, increasing the virus's transmission among the masses (1, 3, 27, 28).

In certain instances, both governments and religious leaders have been criticized for their handling of the pandemic. For example, during the second wave of COVID-19 infections in April 2021 in India, the decision to continue with the annual Kumbh Mela, a pilgrimage that attracts over nine million people, was deemed irresponsible by some public health experts. Despite concerns raised regarding the potential spread of the virus, minimal precautions were taken, and pandemic guidelines were not strictly enforced. These included a lack of social distancing and wearing masks. Such actions have been viewed by some as further contributing to the crisis (21). This led to what some have called as a “massive superspreader event” (45). Unfortunately, no quarantine was enforced nor was contact tracing imposed on the returning pilgrims. This incident highlights how faith and distrust of science can lead to crisis (46). In the Middle East, similar incidents were reported, with increased spread associated in “Qom with Jewish and Shi'ite communities” with religious practices and travel to holy places of their respective countries (23).

In West Africa, the COVID-19 crisis was simply seen by the population as an extension of the Ebola crisis (34). What the Ebola crisis taught may have helped in managing the COVID-19 response, but persisting apocalyptic narratives here also played a role in viral spread (34). When COVID-19 reached Tanzania, that country's president stated that only faith in God and quack treatments like steam inhalation would defend them from COVID-19. He refused to enforce a lock down, and instead rubbished test kits, vaccines, and masking (47).

The pandemic placed many religious and psychospiritual communities in difficult positions, forcing them to make a difficult choice over whether to follow health regulations substantially, partly and not at all, and to keep pursuing their cultural norms, regarding funerals and other sanctified gatherings. In Brooklyn, New York's Hasidic Jewish community, doctors estimated that hundreds of Orthodox Jews died due to participating in super-spreading events such as funerals (48). Funeral restrictions also impacted other religions. Hindus, for example, who commonly cremate the bodies of loved ones in holy sites such as Varanasi, India, have had their travel restricted due to the pandemic. Culturally, these restrictions disrupted an important ritual, one that draws large families together in the throes of cathartic mourning (48). Here, tightly wrapping the bodies of victims of COVID-19 did prevent transmission, but also prevented the victim's families from saying their last goodbyes according to their religious beliefs (48).

Islamic cultures also experienced COVID-19 limitations to their tradition of burying their dead in a timely manner. In Iraq, burials were delayed for days, causing distress among the deceased's loved ones for their inability to provide a traditional funeral (48). The large number of coronavirus deaths also impeded funeral practices, since family members who were recently running from pillar to post to obtain scarce oxygen and a hospital bed, now had to struggle to secure burial plots or space in a funeral home to perform the final rites (49).

Some religious leaders, however, found creative solutions. For example, in the Jewish community, Rabbi Avraham Berkowitz decided to attend a family funeral (and set an example, perhaps) safely distanced in his car (50). Some priests started giving blessings from the hallway or over the telephone, while some funeral homes started drive-in funerals, with others reviving old traditions of bowing to a hearse when it passed by their home. Shivas were organized via video conference by the Jewish community, while Han Chinese live-streamed their tomb-sweeping ceremonies rather than visiting the tomb of their loved ones in person (51). A mixed method review by Burrell et al. (52) revealed that restrictions on funeral practices did not necessarily entail poor outcomes or experiences for the bereaved. Rather, they seemed to add meaning to the occasion and strengthened the connections mourners felt, as they played a much more critical role (52).

Despite these challenges, religious leaders have recognized their unique role in promoting healthy practices, communicating scientific information to their communities, and helping dispel myths and inaccuracies that contributed to the spread of COVID-19. Religious communities have had to encourage people to take precautions, accept vaccinations, adapt, and find innovative ways to continue their practices while minimizing the risk of infection (33, 35, 36, 39–42, 53).

Religion's skepticism over the COVID-19 vaccine coupled with the new technologies being used to practice religion gradually seemed to fade. More in-depth studies found the association between resistance and negative attitude toward vaccination most pronounced in religiously conservative communities (29, 35, 54).

Despite the myriad challenges, several examples appear where religious and community leaders issued guidance based on scientific recommendations and thus adapted their practices in response to the pandemic, changing the implications of these adaptations for public health outcomes. For example: The Catholic Church's Pope Francis loudly and globally professed support for the vaccine (42, 53). While countries with Roman Catholics as the majority religious group displayed a positive association between religiosity and vaccine rates (36). In some regions, religious leaders postponed religious events or utilized alternative modalities to maintain traditions and rituals in a COVID-friendly manner. In Saudi Arabia, the Muslim pilgrimage to the holy sites in the Kingdom were restricted, and a new e-Visa program was devised to ban inbound travel of persons from coronavirus-affected countries (37, 38, 55). In similar fashion, Jewish religious leaders adapted their manner of religious prayer by praying through a “balcony” minyan while conducting online havrutas using video conferencing, and virtually broadcasting Passover ceremonies (33). Programs such as Project Trust have helped religious leaders promote health in ways sensitive to their cultures and provided accurate information about public and mental health during the COVID-19 pandemic (41).

The complex relationship between religiosity, cultural values, and public-health decisions has spurred healthcare professionals and other stakeholders, including religious leadership and policymakers, to examine these factors and formulate effective strategies while promoting cooperation among religious communities to ensure adoption of proper procedures or at least the necessary Standard Operating Procedures (SOPs) to mitigate viral spread (36).

Certainly, religious leaders bear significant influence on the perceptions and behaviors of their followers and congregations, as evidenced by higher country-level religiosity leading to lower vaccination rates. In contrast, Roman Catholics showed the opposite trend, mostly due to the Pope's open advocacy for vaccines. This clearly shows the leveraging role religious leaders can assume in influencing the perceptions and behaviors of their followers in periods of public-health crisis. There is a need for a more-comprehensive approach to science communication, one that considers the needs and perspectives of religious communities in the context of public-health crises (1, 26). As we continue to navigate the challenges of COVID-19 and health crises certain to come, what is essential is considering the role of religious practices and beliefs in either spreading or mitigating the impact of infectious diseases (1, 22–27, 32). Future research should more deeply explore the religion's potential to promote wellbeing and resilience during public-health crises and derive the implications of this for public-health policy and practice (8, 11, 32).

Our discussion considered religion's positive as well as negative impacts during the COVID-19 epidemic. Understanding the complicated relationship between religious practice, belief, and public health is necessary for effective policymaking. It is important to include religious leaders and communities in encouraging compliance to health guidelines, and further, to better understand and promote the role of religion in maintaining wellbeing and resilience during health crises.

As we look ahead to the future, examining our current public health situation raises some pertinent questions. How can religious-based approaches help strengthen adherence to measures such as vaccinations and mask usage? To what degree will technology and virtual platforms impact how people practice religion going forward — is there potential for lasting effects on faith communities and individual believers alike? Also, it's crucial to consider how religion can play a role in shaping public health policies and regulations. This might involve studying how religious organizations and leaders contribute to creating, implementing, and evaluating these policies, and also discovering the most suitable methods to take religious perspectives into account when planning public health initiatives. These are some pressing research topics worth exploring in future to gain deeper insights into ways that religious institutions play critical roles during this pandemic period.

## 6. Conclusion

The COVID-19 pandemic has had a significant impact on people globally for the past 3 years, with vaccine hesitancy greatly hindering efforts to curb its effects. To combat this, collaborative efforts among religious leaders, governments, and scientists remain crucial to building trust and promoting vaccine uptake. It is essential to recognize that effective communication strategies and direct channels are necessary to reach and ease the fears of vaccine-hesitant populations, especially within those communities with strong or strict religious and psychospiritual beliefs. Tailored messaging that is culturally and religiously sensitive has been shown to be more effective in reaching these populations. Religious leaders and institutions can play critical roles in disseminating accurate and evidence-based vaccine information. For instance, faith-based advocacy can help reach certain vaccine-hesitant populations in religious communities. What is important to acknowledge and transcend, however, are the challenges and limitations of such efforts, for political or ideological barriers may exist between certain religious groups and governments that could hinder collaboration.

More generally, governments should concurrently take proactive measures to ensure health safety, equal access to healthcare, and non-discrimination across all communities. Collaborative efforts among all stakeholding groups—religious, scientific, healthcare, and governmental—are necessary to ensure successful vaccination campaigns to curb the spread of pandemics, COVID-19 or those of the future. To this end, it is crucial to survey and provide specific examples of successful collaborations between religious leaders, governments, and scientists to promote vaccine uptake. These should include case studies and real-world examples of faith-based advocacy efforts that have successfully reached vaccine-hesitant populations. Only through such collaborative efforts, can we ensure that all communities receive accurate information and access to vaccinations.

## 7. Limitations

One limitation of this review paper is that it focuses mainly on challenges faced by religious communities during the COVID-19 pandemic without exploring other factors that may contribute to vaccine hesitancy or resistance. This paper also focuses primarily on examples from the Western, Arabs and African contexts, which may limit the generalizability of the findings to other regions or cultural contexts. Additionally, while this paper highlights the importance of engaging religious leaders in promoting vaccination acceptance, it does not explore potential challenges or barriers to such engagement. Our methodology follows a systematic approach, sharing similarities with established guidelines such as PRISMA or Cochrane, but does not strictly adhere to these guidelines. However, our review maintains rigor and transparency, which are key elements of a reliable review process.

## Data availability statement

The original contributions presented in the study are included in the article/supplementary material, further inquiries can be directed to the corresponding author.

## Author contributions

SAh, TB, and GA designed the study and developed the original protocol. SAh and LJ assisted with the initial screening of papers, data extraction, and the literature search. VS, LJ, and SAy contributed to writing the manuscript, including the methods section and synthesis of studies, and also participated in the interpretation of results. LJ, AB, SAy, and NM were involved in data analysis, interpretation of results, and writing several sections of the manuscript. MT, GG, and AB contributed to writing the discussion and conclusion sections. LJ and SAy contributed to writing the results, discussion section, and references. All authors contributed to the article and approved the submitted version.
